# The Glycosylation Status of PrP^C^ Is a Key Factor in Determining Transmissible Spongiform Encephalopathy Transmission between Species

**DOI:** 10.1128/JVI.02296-14

**Published:** 2015-02-11

**Authors:** Frances K. Wiseman, Enrico Cancellotti, Pedro Piccardo, Kayleigh Iremonger, Aileen Boyle, Deborah Brown, James W. Ironside, Jean C. Manson, Abigail B. Diack

**Affiliations:** a Neurobiology Division, The Roslin Institute and R(D)SVS, University of Edinburgh, Easter Bush, United Kingdom; b The National Creutzfeldt-Jakob Disease Research & Surveillance Unit, University of Edinburgh, Edinburgh, United Kingdom; c Food and Drug Administration, Rockville, Maryland, USA

## Abstract

The risk of transmission of transmissible spongiform encephalopathies (TSE) between different species has been notoriously unpredictable because the mechanisms of transmission are not fully understood. A transmission barrier between species often prevents infection of a new host with a TSE agent. Nonetheless, some TSE agents are able to cross this barrier and infect new species, with devastating consequences. The host PrP^C^ misfolds during disease pathogenesis and has a major role in controlling the transmission of agents between species, but sequence compatibility between host and agent PrP^C^ does not fully explain host susceptibility. PrP^C^ is posttranslationally modified by the addition of glycan moieties which have an important role in the infectious process. Here, we show *in vivo* that glycosylation of the host PrP^C^ has a significant impact on the transmission of TSE between different host species. We infected mice carrying different glycosylated forms of PrP^C^ with two human agents (sCJDMM2 and vCJD) and one hamster strain (263K). The absence of glycosylation at both or the first PrP^C^ glycosylation site in the host results in almost complete resistance to disease. The absence of the second site of N-glycan has a dramatic effect on the barrier to transmission between host species, facilitating the transmission of sCJDMM2 to a host normally resistant to this agent. These results highlight glycosylation of PrP^C^ as a key factor in determining the transmission efficiency of TSEs between different species.

**IMPORTANCE** The risks of transmission of TSE between different species are difficult to predict due to a lack of knowledge over the mechanisms of disease transmission; some strains of TSE are able to cross a species barrier, while others do not. The host protein, PrP^C^, plays a major role in disease transmission. PrP^C^ undergoes posttranslational glycosylation, and the addition of these glycans may play a role in disease transmission. We infected mice that express different forms of glycosylated PrP^C^ with three different TSE agents. We demonstrate that changing the glycosylation status of the host can have profound effects on disease transmission, changing host susceptibility and incubation times. Our results show that PrP^C^ glycosylation is a key factor in determining risks of TSE transmission between species.

## INTRODUCTION

Transmissible spongiform encephalopathies (TSE), or prion diseases, are fatal neurodegenerative diseases that can be sporadic, genetic, or acquired by infection (1). These diseases are characterized by a distinct pathology in the central nervous system (CNS), with neuronal loss, spongiform degeneration, and gliosis (2). Numerous mammalian species are susceptible to infection with TSE agents, such as scrapie in sheep and goats, bovine spongiform encephalopathy (BSE) in cattle, Creutzfeldt-Jakob disease (CJD) in humans, and chronic wasting disease (CWD) in cervids.

The host cellular protein PrP^C^ has been shown to have a key role in the transmission of disease (3, 4). During the disease process, PrP^C^ misfolds from the normal conformation to an aberrant form (PrP^Sc^), which is partially resistant to proteases. The prion hypothesis proposes that PrP^Sc^ is the infectious agent responsible for disease transmission and that it is able to self-propagate and induce TSE disease in a new host in the apparent absence of any nucleic acid (5).

Transmission of TSE between different species often is limited by a species barrier to infection (6, 7). In experimental models of disease, the species barrier is characterized by an inefficient primary infection with low susceptibility and long incubation times in the new host. Adaptation to the new host then usually occurs in subsequent passages with an increased attack rate and shorter incubation time (6, 8). In naturally occurring TSE, the species barrier prevents transmission of certain agents between different species. However, some agents have been shown to be able to cross this barrier and cause devastating epidemics in a new host. For example, BSE in cattle can be transmitted to humans via the oral route to cause variant CJD (vCJD) (9, 10). BSE also was able to naturally infect a number of different species, such as goats, nyala, kudu, and domestic or captive wild cats (11–13). Understanding how the species barrier is regulated is important, so that the zoonotic potential of a TSE in other animal populations transmitting to humans can be assessed. This is particularly important for newly emergent strains of TSE in both farmed and wild animals (8, 14).

Despite many studies in recent decades, the mechanisms regulating the species barrier to TSE transmission still are elusive. It has been proposed that sequence identity between host and donor PrP^C^ is important to determine the barrier to transmission. In particular, evidence suggests that sequence homology between host PrP^C^ and PrP^Sc^ leads to high susceptibility and shorter incubation time, whereas sequence differences between these two proteins can lead to lower susceptibility of the host (6, 15, 16). However, this is not always the case (17–19), and it becomes difficult to predict the transmissibility of a strain in a new recipient based solely on sequence identity between host and donor. It is likely that other factors should be taken in account to understand and predict the species barrier.

PrP^C^ is variably glycosylated at two highly conserved sites (amino acid positions 180 and 196 in mice). N-glycan attachment to these sites results in four glycosylated forms (glycotypes) of PrP, diglycosylated, monoglycosylated at position 180, monoglycosylated at position 196, and unglycosylated. While the ratios of diglycosylated, monoglycosylated, and unglycosylated PrP^C^ remain reasonably constant in uninfected brains, the ratios of PrP^Sc^ are highly variable in brains infected with different TSE agents.

*In vitro* studies have demonstrated that the choice of mutation at the glycosylation sites can have profound effects on PrP trafficking, preventing infection of the resulting mutant mice (20). In order to define the role of glycosylation in the transmission of disease, glycosylation-deficient mice have been produced by three laboratories, and inoculations with a number of TSE agents derived from the same species have been carried out (20–22). Conventional transgenic mice expressing hamster PrP in which the first N-glycan site (T183A) or both the first and second site (T183A, T199A) were disrupted were resistant to hamster-passaged prion strains Sc237 and 139H. Mice devoid of the second site also were resistant to 139H but susceptible to Sc237, with extended incubation periods compared to those of the controls (21). In contrast, transgenic mice expressing a 3F4-tagged murine PrP devoid of the first site (T182N) showed no evidence of resistance to any of the murine agents ME7, 139A, and murine-passaged BSE (301C) (20). Mice lacking the second site (T198A) also were susceptible to these strains with extended incubation times (20). Cancellotti et al. produced three inbred lines of gene-targeted mice in which the first (G1; N180T), second (G2; N196T), or both (G3; N180T and N196T) N-glycan attachment sites were disrupted. The homozygous transgenic mice produce partially or unglycosylated PrP^C^ under the control of the endogenous mouse *Prnp* promoter (22). Mice lacking the first N-glycan site were susceptible to 79A, but unlike those of Neuendorf et al., they were resistant to ME7. Mice lacking the second site were susceptible to a number of TSE strains, including ME7, 79A, and 301C, similar to those of Neuendorf et al. However, extended incubation periods were observed for all strains in Neuendorf et al. but only in the case of 301C in Tuzi et al. (20, 23). The differences between the lines of mice can be attributed to many variables, including the different point mutations used to disrupt the glycosylation sites, the species of origin of the PrP gene, different gene constructs, the addition of epitopes, copy numbers, and integration sites, and strain differences of the inocula. The gene-targeted mice, however, remove at least some of the variability observed with the conventional lines of mice.

Multiple strains of TSE agents have been identified. Strains differ in their disease characteristics, such as their transmissibility to other host species or the degree of pathology induced in the brain of the host (24–26). These different strains are proposed to result from different conformations of PrP^Sc^ (27–29). The ratio of the glycotypes of PrP^Sc^ also differs between TSE strains (30, 31). For example, PrP^Sc^ associated with vCJD is predominately diglycosylated, whereas that associated with sporadic CJD (sCJD) is mostly monoglycosylated (32). The N-glycans attached to PrP may influence the conformational flexibility of the protein and could influence its misfolding (33, 34). Conformational flexibility may be particularly important during interspecies transmission of TSE, as the infectious agent must adapt to the novel source of PrP^C^. Indeed, *in vitro* interspecies misfolding of PrP^C^ induced by PrP^Sc^ is inhibited specifically by glycosylation of the protein (35). Moreover, the replication of a given strain may require the misfolding of a precise combination of PrP^C^ glycotypes to replicate the glycoform (36). For example, the replication of a strain in which PrP^Sc^ is predominately monoglycosylated may be favored in a host that produces an elevated level of monoglycosylated PrP^C^.

Here, we have tested the effect of host PrP^C^ glycosylation on the TSE species barrier. This is the first *in vivo* study of the role of glycosylation of PrP^C^ on the transmission of TSE agents between species. Previous *in vitro* and *in vivo* studies to investigate this issue have provided inconsistent results (35, 37, 38) or used mouse-passaged TSE agents (20, 21). Some studies have suggested that the glycosylation of PrP^C^ impedes the transmission between host species (35), whereas others have shown no such effect (20, 38).

We challenged three lines of gene-targeted glycosylation mutants (22) with three nonmurine TSE agents: human vCJD, human sCJD (sCJDMM2), and hamster scrapie 263K. These agents differ in their PrP^Sc^ glycoform ratios and their relative transmissibility to wild-type mice (9, 39, 40). Two of the agents used in this study (263K and vCJD) have similar PrP^Sc^ glycoform ratios in which PrP^Sc^ is predominately diglycosylated. The sCJDMM2 agent has an amino acid sequence identical to that of the vCJD agent and a cleavage site similar to that of proteinase K (PK); however, it has a significantly different PrP^Sc^ glycoform ratio, as the PrP^Sc^ associated with this agent is predominately monoglycosylated. By comparing the relative transmissibility of these TSE agents to our transgenic models, we have established the importance of host PrP^C^ glycosylation in determining the transmissibility of a TSE strain across a species barrier.

## MATERIALS AND METHODS

### Preparation of TSE inocula and intracerebral injection.

Inocula were prepared from brain tissue from a patient with pathologically confirmed sCJDMM2 (0.1% [wt/vol] in physiological saline), the National Institute for Biological Standards and Control (NIBSC) variant CJD reference case (0.1% [wt/vol] in physiological saline), and a hamster with clinical scrapie, strain 263K (1% [wt/vol] in physiological saline). Consent for the use of these materials for research was obtained with ethics approval by the Lothian National Health Service Research Ethics Committee (reference no. 2000/4/157). G1 (N180T), G2 (N196T), and G3 (N180T, N196T) transgenic and wild-type mice were genotyped by mismatched PCR as previously described (22) and coded prior to intracerebral (i.c.) injection with 20 μl of inoculum. All groups were age and sex matched.

The second passage was carried out in a similar manner using brain material from selected G2 and wild-type mice showing evidence of TSE vacuolation and/or PrP deposition. A second passage was not carried out in G1 or G3 mice due to extremely low numbers of mice exhibiting TSE vacuolation and/or PrP deposition. Inocula were prepared from mouse brain tissue at 0.1% (wt/vol) in physiological saline, and mice were inoculated via the i.c. route with 20 μl. Animal experiments were approved by The Roslin Institute's Ethical Review Board and were conducted according to the regulations of the 1986 United Kingdom Home Office Animals (Scientific Procedures) Act.

### Scoring of clinical TSE disease and pathology.

Mice were scored weekly for clinical signs from 100 days postinoculation (dpi) by operators blind to animal genotype according to a previously established TSE clinical scoring system (41). Mice were scored as unaffected, possibly affected, or definitely affected using standard criteria, including kyphosis, ataxia, paralysis, hyperactivity, urinary incontinence, and weight loss. Any unusual clinical signs were noted. In older animals, signs of aging (kyphosis, weight loss, and reduced activity) were taken into account and were classified as possibly affected due to the similar nature of aging and TSE disease phenotypes. Mice were sacrificed after (i) two consecutive scores of definitely affected, (ii) after receiving scores of definitely affected in 2 out of 3 weeks, or (iii) significant deterioration of condition. Mice with no signs of clinical disease were maintained until approximately 700 dpi, at which point the studies were terminated. Animals in which clinical signs were present without pathological (TSE vacuolation and/or PrP deposition) confirmation were removed from the analysis, as these signs also can be due to other conditions, such as aging, as detailed above. Incubation periods were calculated as the number of days between injection and the clinical endpoint in animals with TSE vacuolation. In the absence of an incubation period, the survival time was calculated in days.

Half brains were fixed in formal saline for 48 h and decontaminated with formic acid, when required, prior to paraffin embedding. Coronal sections were cut and stained with hematoxylin and eosin, and TSE-specific vacuolation was semiquantitatively scored blind to TSE strain and mouse genotype by standard methods (42). Vacuolation profiles were plotted for groups of 6 mice or more. Genotypes of all mice were confirmed postmortem by PCR (22). Early intercurrent deaths (under 200 dpi for the first pass and 100 dpi for the second pass) were excluded from the study.

### Biochemical assessment of PrP^Sc^ in first-pass mice.

Half brains from mice challenged with either sCJDMM2, vCJD, or 263K were used to prepare a 1% (wt/vol) inocula in physiological saline; from this, PrP^Sc^ was extracted using extraction buffer (NP-40, sodium deoxycholate, sodium chloride, Tris [pH 7.4]) and digested with PK (37°C for 1 h, 20 μg/ml). Reactions were terminated by the addition of phenylmethylsulfonyl fluoride. If required, PrP^Sc^ then was concentrated by sodium phosphotungstic acid (NaPTA)-mediated precipitation (43). Samples (brain homogenate/NaPTA-concentrated PrP^Sc^) were analyzed for PrP^Sc^ by Western blotting using the anti-PrP antibody 8H4 (1/10,000) (44). PrP^Sc^ could not be easily detected in vCJD-challenged G2 mice. In order to detect PrP^Sc^ in G2 mice, NaPTA-mediated precipitation was carried out on 10% (wt/vol) brain homogenates such that the equivalent of 5 μg of total brain was run per G2 well. Equal PrP signal then was achieved by diluting wild-type PrP^Sc^ 10-fold prior to electrophoresis.

### Immunohistochemistry.

Coronal brain sections were stained using the 6H4 antibody (1/20,000; Prionics) to detect PrP. Antigen retrieval by autoclaving at 121°C for 15 min and a 5-min formic acid (98%) treatment was used. Sections then were blocked with normal rabbit serum prior to incubation with the primary antibody. Antibody binding was detected with either the catalyzed signal amplification system (Dako) or Vector ABC kit (Vector Laboratories) and visualized with 3,3′-diaminobenzidine chromogen. All sections were counterstained with hematoxylin. In all experiments, normal brain homogenate-inoculated mouse and PrP^−/−^ mouse (NPU, Edinburgh, United Kingdom) sections were used as a negative control (4).

### Biochemical assessment of PrP^C^ in uninfected glycosylation-deficient transgenic mice.

Brains from uninfected G1, G2, G3, and wild-type mice were used to prepare 10% (wt/vol) homogenates. An α-tubulin loading control was included to determine variation in the amount of protein being loaded. Samples were analyzed by Western blotting using seven primary anti-PrP antibodies (7A12/epitope, conformation epitope; DE3/epitope, aa146-153; FH10/epitope, aa202-210; AE11/epitope, aa140-145; BH1/epitope, aa143/154; FD12/epitope, unknown at 0.1 μg/ml; and BC6/epitope, aa146-156 at 0.01 mg/ml) (45, 46) and species-specific peroxidase-conjugated AffiniPure antibodies (Stratech) at 0.05 μg/ml diluted in 0.5% blocking solution for 30 min at room temperature. Bound secondary antibody was detected by light emission from SuperSignal West Dura chemiluminescent substrate using a Kodak 440 Image Station with typical exposures of 1, 5, and 10 min. Immunoblots were imaged using the Kodak Image Station and Kodak MI software. Protein bands were selected manually by drawing around them and the sum intensity calculated. A blank region also was selected to measure the background intensity of the blot.

## RESULTS

### Absence of the first and both PrP^C^ glycosylation sites limits transmission with all three agents.

Infection of G1 and G3 mice with vCJD and sCJDMM2 resulted in no pathologically confirmed clinical disease and no evidence of TSE vacuolation and/or PrP deposition in the brain (Tables 1 and 2). While there were a number of G1 mice (4/14) with TSE vacuolation and/or PrP deposition after 263K infection, again there were no cases of clinical disease or TSE vacuolation and/or PrP deposition in G3 mice (Table 3). Thus, the absence of either the first or both PrP^C^ glycosylation sites in the host appears, particularly in the case of vCJD and sCJDMM2, to restrict replication of the agent within the CNS compared to that of the wild-type mice.

**TABLE 1:** Sporadic CJDMM2 inoculation into glycosylation-deficient mice at first and second^*a*^ passages

Mouse line	Inoculum	Incubation time^*b*^ (days) ± SEM (range)	Clinical disease (no. positive/total no.)	TSE vac and/or PrP deposition^*c*^	Survival time (days) of mice with TSE vac and/or PrP^*d*^
G1	sCJDMM2		0/18	0/18	
G2	sCJDMM2	404 ± 8 (378–430)	7/20	11/20	367 ± 50 (245–494)
G3	sCJDMM2		0/18	0/18	
Wt	sCJDMM2		0/41	3/41	658 ± 49 (559–707)
G2	G2sCJDa	129 ± 3	11/11	11/11	
Wt	G2sCJDa	430 ± 20	12/12	12/12	
G2	G2sCJDb	147 ± 3	10/11	11/11	
Wt	G2sCJDb	439 ± 22	10/10	10/10	

a Brain material from two sCJDMM2-inoculated G2 mice with pathological evidence of disease were further passaged into G2 and wild-type mice.

b Incubation time for mice with clinical signs and evidence of TSE vacuolation. Studies were maintained to ∼700 dpi for mice with no clinical signs of TSE disease.

c TSE vacuolation (vac) and/or PrP deposition were used as evidence of transmission for primary passage due to low numbers of mice with clinical signs and vacuolation present. Limited confirmatory immunohistochemistry for PrP deposition was carried out in the second passage due to high numbers of mice with clinical signs and vacuolation present.

d Survival time of mice with no clinical signs and evidence of vacuolation and/or PrP deposition.

**TABLE 2:** Variant CJD inoculation into glycosylation-deficient mice at first and second^*a*^ passages

Mouse line	Inoculum	Incubation time^*b*^ (days) ± SEM (range)	Clinical disease (no. positive/total no.)	TSE vac and/or PrP deposition^*c*^	Survival time (days) of mice with TSE vac and/or PrP^*d*^
G1	vCJD		0/17	0/17	
G2	vCJD	536 ± 16 (510–600)	8/18	14/18	611 ± 29 (505–708)
G3	vCJD		0/19	0/19	
Wt	vCJD	477 ± 15 (380–616)	21/40	36/40	483 ± 18 (310–616)
G2	G2vCJDa	283 ± 3	10/11	11/11	
Wt	G2vCJDa	192 ± 2	12/12	12/12	
G2	G2vCJDb	315 ± 3	11/12	12/12	
Wt	G2vCJDb	193 ± 3	12/12	12/12	
G2	WtvCJD	334 ± 6	11/13	13/13	
Wt	WtvCJD	156 ± 1	12/12	12/12	

a Brain material from two vCJD-inoculated G2 mice and one vCJD inoculated wild-type mouse with pathological evidence of disease were further passaged into G2 and wild-type mice.

b Incubation time for mice with clinical signs and evidence of TSE vacuolation. Studies were maintained to ∼700 dpi for mice with no clinical signs of TSE disease.

c TSE vacuolation and/or PrP deposition were used as evidence of transmission for primary passage due to low numbers of mice with clinical signs and vacuolation present. Limited confirmatory immunohistochemistry for PrP deposition was carried out in the second passage due to high numbers of mice with clinical signs and vacuolation present.

d Survival time of mice with no clinical signs and evidence of TSE vacuolation and/or PrP deposition.

**TABLE 3:** 263K inoculation into glycosylation-deficient mice at first and second passages^*a*^

Mouse line	Inoculum	Incubation time^*b*^ (days) ± SEM (range)	Clinical disease (no. positive/total no.)	TSE vac and/or PrP deposition^*c*^	Survival time (days) of mice with TSE vac and/or PrP^*d*^
G1	263K		0/14	4/14	579 ± 35 (507–674)
G2	263K		0/18	11/18	552 ± 18 (461–629)
G3	263K		0/16	0/16	
Wt	263K		0/18	11/18	612 ± 18 (503–698)
G2	G2–263Ka	284 ± 7	12/12	12/12	
Wt	G2–263Ka	437 ± 32	10/12	9/12	
G2	G2–263Kb	320 ± 7	11/11	11/11	
Wt	G2/263Kb	536 ± 6	4/10	6/10	343 ± 61 (282–408)
G2	Wt263K	365 ± 0	11/12	12/12	
Wt	Wt263K	172 ± 5	9/10	10/10	

a Brain material from two 263K-inoculated G2 mice and one 263K-inoculated wild-type mouse with pathological evidence of disease were further passaged into G2 and wild type mice

b Incubation time for mice with clinical signs and evidence of TSE vacuolation. Studies were maintained to ∼700 dpi for mice with no clinical signs of TSE disease.

c TSE vacuolation and/or PrP deposition were used as evidence of transmission for primary passage due to low numbers of mice with clinical signs and vacuolation present. Limited confirmatory immunohistochemistry for PrP deposition was carried out in the second passage due to high numbers of mice with clinical signs and vacuolation present.

d Survival time of mice with no clinical signs and evidence of TSE vacuolation and/or PrP deposition.

### Absence of sugars at the second glycosylation site of PrP^C^ in the host removes the barrier to infection with sCJD.

**FIG 1:**
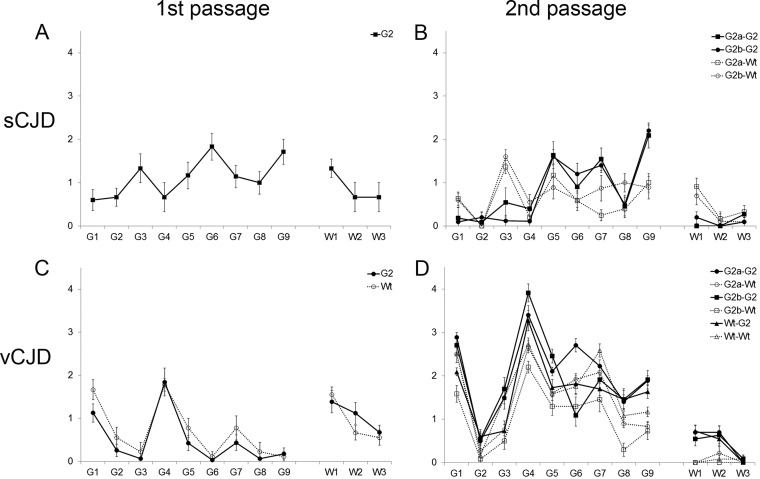
Lesion profile analysis of wild-type (Wt) and G2 mice after intracerebral inoculation with sCJDMM2 (A), second passage of sCJDMM2 (B), vCJD (C), and second passage of vCJD (D). The second passage was carried out from selected G2 and wild-type mice showing TSE vacuolation and/or PrP. Group size, *n* ≥ 6 (± standard errors of the means). Gray matter scoring regions are labeled G1 to G9: G1, dorsal medulla; G2, cerebellar cortex; G3, superior colliculus; G4, hypothalamus; G5, thalamus; G6, hippocampus; G7, septum; G8, retrosplenial cortex; G9, cingulate and adjacent motor cortex. White-matter scoring regions are labeled W1 to W3: W1, cerebellar white matter; W2, mesencephalic tegmentum; W3, pyramidal tract.

**FIG 2:**
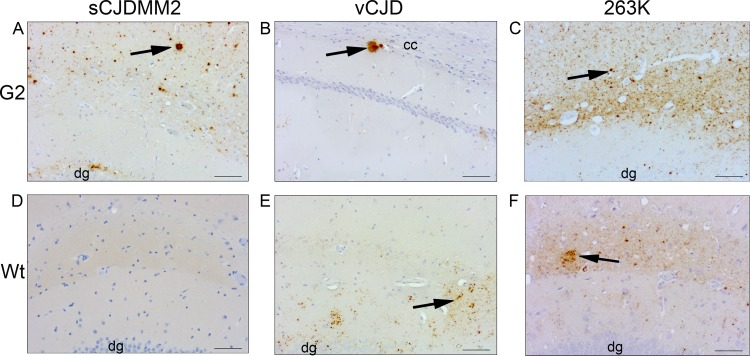
PrP deposition in the brains of wild-type and G2 mice after intracerebral inoculation with sCJDMM2, vCJD, or 263K agent. (A) G2 mouse inoculated with sCJDMM2; (B) G2 mouse inoculated with vCJD; (C) G2 mouse inoculated with 263K; (D) wild-type mouse inoculated with sCJDMM2; (E) wild-type mouse inoculated with vCJD; (F) wild-type mouse inoculated with 263K. Arrows indicate examples of PrP accumulation in the form of plaque-like deposits in panels A and B and examples of fine punctate PrP accumulation in panels C, E, and F. No PrP accumulation was detected in panel D. PrP was detected with 6H4 antibody. dg, dentate gyrus; cc, corpus callosum. Scale bars, 500 μm.

**FIG 3:**
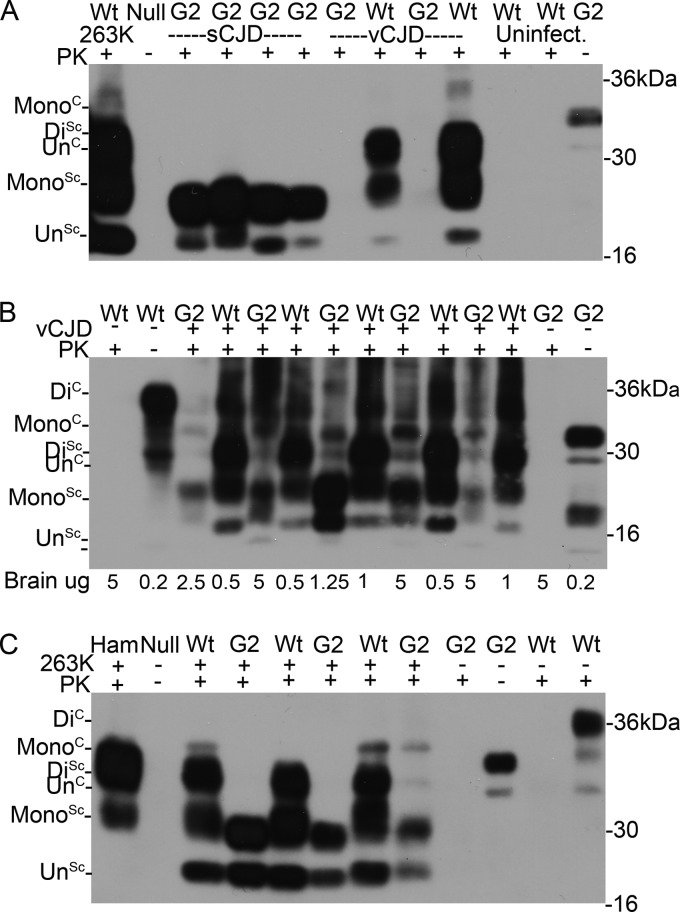
Biochemical analysis of PrP^Sc^ from the brains of G2 and wild-type (Wt) mice inoculated with sCJDMM2, vCJD, and 263K. (A) PrP^Sc^ was isolated from four sCJD-challenged G2 mice, one vCJD-challenged G2 mouse (assayed in duplicate), and one vCJD-challenged wild-type mouse (assayed in duplicate) by standard PK digestion. PrP^Sc^ was concentrated by NaPTA precipitation. The amount of PrP^Sc^ in the vCJD-challenged G2 sample was too low to detect. Uninfected mice and a 263K-challenged wild-type mouse were included as controls. (B) PrP^Sc^ was isolated from vCJD-challenged G2 mice by standard PK digestion followed by NaPTA precipitation. The amount of PrP^Sc^ was equalized by dilution following NaPTA precipitation to allow comparison (equivalent of 5 μg of G2 brain and 0.5 to 1 μg of wild-type brain). Uninfected mice were included as controls. (C) PrP^Sc^ was isolated from three 263K-challenged G2 mice and three wild-type mice by standard PK digestion. Uninfected mice and a 263K-challenged hamster were included as controls. The different isoforms of PrP are denoted Di (for diglycosylated), Mono (monoglycosylated), and Un (unglycosylated). PrP^Sc^ was detected with 8H4.

G2 transgenic mice were susceptible to sCJDMM2, in contrast to the G1, G3, and wild-type mice (Table 1; also see Fig. 2D). Seven out of 20 of the sCJDMM2-challenged G2 transgenic mice developed clinical disease with an average incubation time of 404 ± 8 days (Table 1). Disease status was confirmed by pathology within the brain in which both TSE vacuolation and PrP deposition were observed (Fig. 1A and 2A). PK-resistant PrP^Sc^ also was detected by Western blotting in brains of these mice (Fig. 3A). The molecular weight of PrP^Sc^ in these samples is consistent with the majority of PrP^Sc^ being monoglycosylated, as observed in previous studies using these mice (23). Thus, removal of the second glycosylation site of the host PrP^C^ has rendered the host susceptible to cross-species transmission with sCJDMM2.

Brain material from two G2 mice that had developed clinical disease following inoculation with sCJDMM2 (G2-sCJD) was used to challenge G2 and wild-type mice. All challenged G2 mice rapidly developed a clinical disease with remarkably short incubation times of 129 ± 3 days and 147 ± 3 days (Table 1). Wild-type mice that previously were resistant to infection with the sCJDMM2 agent developed a clinical disease after challenge with G2-sCJD brain; however, in this case the incubation time was much longer than that for G2 recipients (430 ± 20 days and 439 ± 22 days) (Table 1).

The pathological signs of disease differed in the two hosts, with the vacuolation profile in G2 mice distinct from that observed in wild-type mice (Fig. 1B). The intensity of TSE vacuolation in the superior colliculus was lower in G2 mice than in wild-type animals infected with G2-sCJD brain material. More intense TSE vacuolation occurred in the cingulate cortex of the G2 mice than in wild-type animals after challenge with G2-sCJD brain material (Fig. 1B). Primary and secondary passage of sCJDMM2 in G2 mice showed a similar trend in vacuolation intensity throughout the brain (Fig. 1A and B). The lack of the second glycosylation site in the host appears to greatly facilitate the infection with sCJD, suggesting that the diglycosylated form of host PrP is important in maintaining the species barrier of a predominantly monoglycosylated strain.

### Diglycosylated PrP^C^ in the host influences incubation time in the interspecies transmission of vCJD.

Variant CJD PrP^Sc^ has an identical amino acid sequence and PK cleavage pattern by Western blotting similar to that of PrP^Sc^ from sCJDMM2 but is predominately diglycosylated compared with the predominantly monoglycosylated sCJDMM2. Moreover, vCJD has been shown to transmit readily to wild-type mice, in contrast to sCJDMM2. Twenty-one out of 40 wild-type mice challenged with vCJD developed clinical disease with an average incubation time of 477 ± 15 days. A similar proportion of G2 mice (8/18) developed clinical disease, with an average incubation time of 536 ± 16 days (Table 2).

The pattern of TSE vacuolation was similar in G2 transgenic and wild-type mice infected with vCJD (Fig. 1C). However, as shown in Fig. 2B and E, there is less PrP deposition in the brains of animals expressing monoglycosylated PrP (G2) than in those of the wild-type mice. Diffuse deposition of PrP was observed in the brains of wild-type mice. In contrast, small plaque-like deposits of PrP were observed in the brains of G2 transgenic mice. In order to detect PrP^Sc^ in the G2 mouse brain, NaPTA precipitation of PrP^Sc^ was required, followed by equalized PrP^Sc^ loading (5 μg of G2 brain versus 1 μg of wild-type brain), indicating that there was less PrP^Sc^ in G2 mice than in wild-type mice (Fig. 3B).

To further investigate the effect of the second site on vCJD transmission, we inoculated brain material from two G2 mice (G2-vCJD with monoglycosylated PrP^Sc^) and a wild-type mouse (Wt-vCJD with fully glycosylated PrP^Sc^) previously infected with vCJD into G2 and wild-type recipients. Wild-type mice expressing fully glycosylated PrP^C^ developed clinical disease within 192 ± 2 days and 193 ± 3 days of infection with two independent G2-vCJD isolates. Two groups of G2 mice that were challenged with these isolates developed clinical disease later than the wild-type controls (283 ± 3 days and 315 ± 3) (Table 2). The same pattern also was observed when mice were challenged with the Wt-vCJD brain material; G2 mice developed a clinical disease later (334 ± 6 days) than wild-type mice (156 ± 1 days) (Table 2). Despite changes in incubation times, the pattern of TSE vacuolation appeared to be similar between G2 and wild-type mice and between primary and secondary passages of vCJD (Fig. 1C and D).

### Glycosylation compatibility between the hamster strain 263K and host PrP^C^ reduces the species barrier.

Primary transmission was carried out with the hamster TSE strain 263K. PrP^Sc^ associated with the 263K agent is predominately diglycosylated. In contrast to vCJD, wild-type mice do not develop a clinical disease with this TSE strain but do show evidence of PK-resistant PrP^Sc^ in the brain following inoculation (39, 47). After challenge with 263K, no wild-type or G2 mice exhibited clinical disease, consistent with previous data. However, both G2 and wild-type mice demonstrated pathological signs of a subclinical infection (G2, 11/18; wild type, 11/18) (Table 3 and Fig. 2C and F). PK-resistant PrP^Sc^ also was detected in the brains of these mice by Western blotting (Fig. 3C). The PrP^Sc^ glycoprofile in these samples is consistent with the majority of PrP^Sc^ being monoglycosylated in G2 recipients and fully glycosylated in wild types.

G2 and wild-type mice were challenged by an i.c. route with brain homogenate from two 263K-inoculated G2 mice (G2-263K with monoglycosylated PrP^Sc^) and a 263K-infected wild-type mouse (Wt-263K with fully glycosylated PrP^Sc^). In contrast to the vCJD transmissions, G2 mice had a higher susceptibility to disease after challenge with G2-263K than wild-type mice; all G2 mice succumbed to disease after challenge, whereas 10/12 (with the first G2 brain) and 4/10 (with the second G2 brain) wild-type mice developed clinical signs of TSE disease after G2-263K infection (Table 3). Moreover, G2 mice developed disease more quickly (284 ± 7 days and 320 ± 7 days) than wild-type mice (437 ± 32 days and 536 ± 6 days) after challenge with G2-263K (Table 3). The differences in the attack rate and incubation periods elicited by the two isolates of G2-263K may occur because the two animals from which they were derived were at different stages of subclinical infection; hence, they had different titers of infectivity within their brains. The G2 mice challenged with Wt-263K developed disease later (365 ± 0 days) than wild-type mice (172 ± 5 days) (Table 3). Thus, 263K transmits with similar efficiency in the G2 mice and the wild-type mice in the primary passage, but unlike vCJD on the second passage following G2 transmission, this agent was considerably faster in the G2 host than in the wild-type host.

### PrP^C^ expression is reduced in glycosylation-deficient mice.

**FIG 4:**
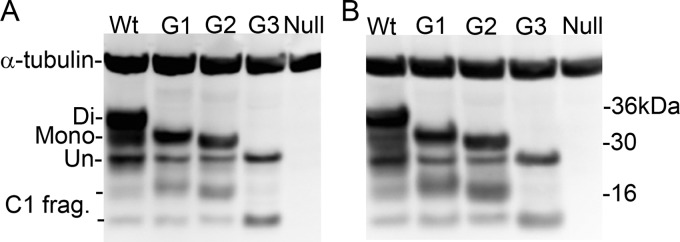
PrP^C^ expression levels in the brains of glycosylation-deficient transgenic mice. Representative Western blots showing the different PrP^C^ isoforms in wild-type (Wt), G1, G2, and G3 mice using BC6 (A) and BH1 (B) antibodies. Western blots underwent densitometry to measure levels of PrP^C^. α-Tubulin was used as a loading control. The different isoforms of PrP^C^ are denoted Di (for diglycosylated), Mono (monoglycosylated), Un (unglycosylated), and C1 frag. (C1 fragments).

To ascertain whether the protein levels of PrP^C^ in any of the lines of mice could influence the incubation times in the transmissions described above, we undertook analysis of the PrP^C^ levels in all mouse lines. Immunoblot analysis showed the expected banding patterns for both the transgenic and wild-type mice. Truncated PrP^C^, designated C1, was observed in all mice and was included in the measurement of total PrP^C^ (selected antibodies are shown in Fig. 4A and B). All of the glycosylation-deficient mice expressed significantly less PrP^C^ than wild-type mice (*P* < 0.0001). G1 and G2 mice expressed approximately 50% and G3 mice only 32% of that found in wild-type mice, with G3 mice expressing significantly less (*P* < 0.01) than both G1 and G2 mice. There was no significant difference in total PrP in each mouse line using different antibodies (Fig. 4A and B).

## DISCUSSION

Expression of PrP^C^ is known to influence incubation times of a TSE disease, with reduced levels of the protein resulting in longer incubation periods (48). Earlier studies showed conflicting results over whether PrP^C^ expression levels are altered within the glycosylation transgenic mice (22). This most likely was due to the epitope recognition of the antibodies used and detection of only a subset of isoforms. Our expanded studies here, using a range of monoclonal antibodies within the C-terminal and central region of PrP^C^, are able to detect all isoforms of PrP^C^, demonstrating that G1, G2, and G3 mice do have lower levels of PrP^C^ expression than wild-type mice. However, while lower levels of PrP^C^ in the G1 and G3 mice may contribute to longer incubation times, the levels observed in these mice are not likely to explain the resistance to TSE disease observed here. Studies have shown that mice heterozygous for PrP^C^ expression and with a level of PrP^C^ expression similar to that of the G1 mice are fully susceptible to TSE disease, albeit with incubation periods of almost twice that of wild-type mice (48–50). Our studies were maintained to approximately 700 dpi, almost twice the incubation period of sCJD in G2 mice, which also show 50% PrP^C^ expression. Thus, factors other than a reduction in PrP^C^ expression level are likely to contribute to the resistance of these mice to TSE disease. While the lower expression of PrP^C^ in G2 mice may contribute to the longer incubation period observed in this model after challenge with vCJD, the G2 mice are more susceptible to infection with the sCJDMM2- and G2-passaged 263K TSE agents despite expressing lower levels of PrP^C^ than wild-type controls. Therefore, this enhanced susceptibility can be directly attributed to the altered glycosylation status of the host.

The monoglycosylated sCJDMM2 agent was transmitted to a normally resistant host (51) by removal of the glycans at PrP residue 196 (as removed in G2 mice). Moreover, sCJDMM2 became adapted in the G2 host and produced very short incubation times on the second pass. The data suggest that the presence of glycans at PrP residue 196 (as present in G1 or wild-type mice) is responsible for the sCJDMM2 transmission barrier; removal of this site may facilitate the interaction between host monoglycosylated PrP^C^ and the infective monoglycosylated PrP^Sc^, allowing replication of the infective agent. This is the first time that glycosylation-deficient transgenic mice have shown an enhanced susceptibility to TSE infection compared to that of wild-type mice. This suggests that glycosylation at the second glycosylation site can protect against transmission both between and within species.

Experimental transmissions from wild-type or G2 mice infected with the 263K strain provide additional evidence that similar glycosylation statuses of host PrP^C^ and the PrP^Sc^ in the inoculated strain can greatly accelerate TSE incubation periods. Indeed, the incubation period in G2 recipients was almost half that of wild-type mice after challenge with the G2-263K strain.

In both primary and secondary passages of vCJD, incubation periods were shorter in wild-type mice than in mice in which the second PrP^C^ N-glycan attachment site was disrupted. The shorter incubation periods were observed irrespective of the glycosylation status of the second site in the infecting PrP^Sc^. While these differences in incubation time can be explained on the basis of lower PrP^C^ expression levels in the G2 mice, we cannot discount the possibility that it indicates a preference of this strain for a PrP^C^ diglycosylated host irrespective of the passage history of the strain. This may explain the ability of this agent to infect a large number of host species and its transmissibility across many species barriers.

G1 and G3 mice showed little susceptibility to infection throughout this study. Indeed, these transgenic mice did not develop any pathologically confirmed clinical TSE disease after inoculation with any of the three agents used, although asymptomatic infection in the form of PrP deposition was detected in extremely low numbers. This may be linked to an inability of this particular host PrP^C^ to propagate nonmurine strains; previous experiments performed with a number of mouse-adapted scrapie strains by several routes have highlighted an intrinsic resistance of both G1 and G3 mice to infection (23, 52). Therefore, it is more likely that the resistance observed in G1 and G3 mice in this study is linked to a more general mechanism rather than an effect of the species barrier. Why the absence of the first glycosylation site should lead to such a dramatic loss of host susceptibility may be related to the conversion efficiency of PrP^C^ to PrP^Sc^. Some *in vitro* conversion assays have previously suggested that glycosylation inhibits the conversion activity (30). However, such *in vitro* systems have not revealed the complexity of the glycosylation issue observed in these *in vivo* studies. The resistance observed in the G3 mice likely is related to the absence of the G1 glycosylation. However, G3 mice also show more C1-truncated PrP^C^ upon biochemical analysis than G1, G2, and wild-type mice. Previous *in vitro* studies have shown that higher levels of C1 PrP^C^ are associated with resistance to TSE infection (53). In addition, G3 mice show the lowest PrP^C^ expression of the three glycosylation mutants and a different PrP^C^ localization (22). All of these factors might contribute to the resistance to TSE infection of this specific line of mice.

The absence of glycans at the second site may alter the biology of PrP^C^ or PrP^Sc^ interaction in a very different way than that of the first glycosylation site. A number of biochemical properties and the cellular localization of PrP^C^ in the G2 mice resemble that observed in wild-type and G1 mice (22). However, the presence/absence of carbohydrates in a specific portion of PrP^C^ may influence other characteristics, such as the ultrastructural localization of PrP^C^ (e.g., localization in a different portion of the cell membrane) or its conformation, and this may dictate the different susceptibility to infection of the G2 mice compared to that of the G1 mice.

We have argued that altered glycosylation status of PrP^C^ alters the host susceptibility. An alternative explanation is that the point mutations inserted in order to modify the N-linked glycosylation sites on PrP are the cause of this change (22). Previous transmission studies performed by us (23) and Neuendorf et al. (20) have shown similar results upon primary passage of both ME7 and mouse BSE strains with prolonged incubation periods in mice deficient at the first glycosylation site despite utilizing different amino acid substitutions and expressing different levels of PrP. In addition, Ikeda et al. (54) showed that substitution of Asn residues to abolish glycosylation sites does not prevent conversion of PrP^C^ to PrP^Sc^. In this study, the differences between the wild-type and G2 hosts in susceptibility to primary passage with two human agents, vCJD and sCJDMM2 (characterized by an identical PrP^Sc^ sequence and PK cleavage pattern but a different glycoprofile), further argues for the glycosylation status being the main determinant of host susceptibility rather than the change in amino acid sequence.

The deposition of PrP in the brains of G2 mice infected with vCJD differed from that observed in wild-type mice infected with the same agent. First, the total amount of PrP that accumulated by disease endpoint appeared to be lower in G2 mice. This could be due to less PrP^C^ being available for replication, or it could mean that the rates of misfolding, clearance, and/or toxicity of PrP are changed in the absence of glycosylation at the second site. In addition, small PrP aggregates were observed in G2 mice infected with vCJD, in contrast to the diffuse PrP deposition observed in wild-type mice. Large aggregated deposits of PrP also were observed in G2 mice challenged with sCJDMM2. These data suggest that PrP^C^ that lacks the second glycosylation site has altered misfolding or clearance kinetics, which also may have an important effect on host susceptibility.

In summary, we propose that the transmission of TSE agents across different species can be profoundly influenced by posttranslational events in both PrP^C^ and PrP^Sc^. In particular, we have demonstrated that the glycosylation status of host PrP^C^ (55, 56) can dramatically alter cross-species transmission characteristics and likely is important for this protein to act as a receptor for the incoming TSE agent.

On the other hand, the prevalence of certain PrP^Sc^ glycotypes in an infectious inoculum may determine its conformation and the ability to interact with the host and cause a TSE infection. This combination may lead to the binding between PrP^Sc^ and PrP^C^ occurring through direct interactions between the glycan residues and/or different PrP regions that have been recently suggested to be important for TSE transmission between different species (57) or by interactions with a number of conversion cofactors previously suggested, such as host proteins or nucleic acids (58–60).

The dramatic effects in altered host susceptibility, in particular the resistance of the G1 and G3 mice to infection, suggests this mechanism provides an important focus for blocking the disease process and protecting the infected individual from neurodegeneration.
